# An efficient non-iterative smoothed particle hydrodynamics fluid simulation method with variable smoothing length

**DOI:** 10.1186/s42492-022-00128-x

**Published:** 2023-01-03

**Authors:** Min Li, Hongshu Li, Weiliang Meng, Jian Zhu, Gary Zhang

**Affiliations:** 1 The School of Computer Science, Guangdong University of Technology, Guangzhou, 510006 China; 2 The School of Information Engineering, Guangdong University of Technology, Guangzhou, 510006 China; 3 The School of Advanced Manufacturing, Guangdong University of Technology, Guangzhou, 510006 China; 4 National Laboratory of Pattern Recognition, Institute of Automation of the Chinese Academy of Sciences, Beijing, 100190 China

**Keywords:** Smoothed particle hydrodynamics, Variable smooth length, Fluid simulation

## Abstract

In classical smoothed particle hydrodynamics (SPH) fluid simulation approaches, the smoothing length of Lagrangian particles is typically constant. One major disadvantage is the lack of adaptiveness, which may compromise accuracy in fluid regions such as splashes and surfaces. Attempts to address this problem used variable smoothing lengths. Yet the existing methods are computationally complex and non-efficient, because the smoothing length is typically calculated using iterative optimization. Here, we propose an efficient non-iterative SPH fluid simulation method with variable smoothing length (VSLSPH). VSLSPH correlates the smoothing length to the density change, and adaptively adjusts the smoothing length of particles with high accuracy and low computational cost, enabling large time steps. Our experimental results demonstrate the advantages of the VSLSPH approach in terms of its simulation accuracy and efficiency.

## Introduction

Fluid simulations are important in computer graphics. Physics-based fluid simulation technologies, such as smoothed particle hydrodynamics (SPH) [1, 2], have been widely applied in feature films and computer games. As a Lagrangian particle-based method, SPH discretely samples a fluid into a group of particles. The physical quantities (e.g., density and velocity) at any location are interpolated using the neighboring fluid particles within a given distance. By storing the information about the particles’ physical quantities, computational resources focus on relevant fluid regions; hence, the simulation efficiency of SPH is usually higher than that of Eulerian grid-based methods [3, 4]. In SPH, the accuracy of interpolation is important for maintaining the stability and realism of the corresponding fluid simulations.

One of the main factors affecting the SPH interpolation accuracy is the smoothing length (i.e., the support radius of the smoothing kernel), which determines the number of neighboring particles involved in the interpolation. It is clear that the interpolation accuracy decreases when there are too few neighbors. Increasing the number of neighbors improves the accuracy, but at the cost of an exponential increase in the computational overhead. For these reasons, a constant support radius with an appropriate length (usually two to four times the particle radius) is usually chosen for these simulations [1, 5, 6].

However, a constant smoothing length has its own disadvantages owing to the lack of adaptiveness. Particles at splashes usually have fewer neighbors than those in steady regions, for example, places where the fluid is steady and far away from the free surface, when the smoothing length is fixed. An opposite situation arises in regions where the fluid motion is quite violent and particles are compressed. Both these situations destabilize simulations, reducing their accuracy.

Some attempts have been made to address the above problem using iterative optimization methods for determining optimal solutions, such as PCISPH [7], IISPH [8], and DFSPH [9, 10]. However, these iterative methods usually incur high computational costs. Variable smoothing-length methods have also been proposed [11] for addressing the adaptation problem, but interpolation processes have been iteration-based, negatively affecting the simulation efficiency.

Leveraging the relationship between the particles’ density and smoothing length, in this study, we propose a variable smoothing-length method with a non-iterative solution. By adaptively adjusting the smoothing length of fluid particles, the neighbors of each particle can be kept stable; hence, the interpolation accuracy is usually higher than that of the previously proposed constant smoothing-length methods. The proposed non-iterative equation is computationally more efficient than iteration-based methods. With higher accuracy owing to the variable smoothing length, the simulation efficiency is further improved, owing to larger time steps.

In summary, the proposed non-iteration-based variable smoothing-length method contains the following contributions.

1. A variable smoothing-length update scheme based on the particles’ density variation is proposed, which does not need to be solved by iterative optimization, ensuring high computational efficiency.
2. A symmetric interpolation kernel is used to ensure the force symmetry between particles with variable smoothing length, guaranteeing the method’s numerical stability.
3. The smoothing length-update scheme is validated in extensive experiments, and its advantages are demonstrated by comparison with several state-of-the-art methods.

### Related work

#### Stability and efficiency of SPH

An improvement of the SPH computational accuracy is typically accompanied by an improvement of the corresponding simulation stability [12, 13]. The improved simulation stability affords larger simulation time steps, thus improving the simulation efficiency. In computer graphics, many classical SPH algorithms have been improved toward this objective.

Researchers have used various methods to improve the interpolation accuracy for calculating physical quantities such as density and force. Density is an important physical parameter in simulations. Many studies in fluid simulations have attempted to modify the density changes of fluids to ensure the fluids’ incompressibility. Among them, the classical PCISPH method [7] corrects the displacement of particles using prediction and correction operations, yielding more stable particle density changes compared with the simple WCSPH method [14] (which uses the Tait equation for direct pressure computation). The PCISPH method affords several-fold longer time steps than the WCSPH method, owing to its better simulation stability. The IISPH method [8] obtains a more accurate pressure field by solving the Poisson equation to maintain the density field as constant as possible. Compared with the WCSPH method, the IISPH method has a more uniform density field, higher incompressibility, and higher simulation stability. The DFSPH method [9, 10] improves the stability and efficiency of the simulation process by constructing velocity fields without divergence or density changes. Wu et al. [15] further improved the DFSPH method using the SOR method, to reduce the number of iterations for the pressure solver. In the above algorithms, iterative calculation processes are used to compute the physical quantities of interest. Although these algorithms allow increasing the time step to improve the simulation efficiency, the efficiency of these single-step simulations is significantly lower than that of the WCSPH method.

Yang et al. [16] proposed a pairwise force smoothing particle hydrodynamics model with a larger support radius than the standard SPH method and an anisotropic filtering term, to avoid particle aggregation on free surfaces, for improving the simulations’ stability. Weiler et al. [17] proposed an implicit viscosity solver based on physical continuity, which outperformed previous methods in terms of physical accuracy and memory consumption, thus enabling high-resolution complex fluid animations. Using a volumetric-centered SPH discretization method, Band et al. [18] proposed a boundary pressure-treatment method for the IISPH method that effectively reduced pressure oscillations at the boundary and afforded large time steps. Bender et al. [19, 20] proposed a boundary processing method based on density maps, which improved the stability and authenticity of simulations. Gissler et al. [21] proposed a chain-type SPH pressure solver for strong fluid-structure interactions, which stabilized the processing of the fluid-structure interface and significantly reduced the computational overhead by affording larger time steps.

#### Adaptivity in SPH

The main objectives of all fluid simulations are to improve the simulations’ quality and efficiency. However, the two are often contradictory and difficult to ensure simultaneously; thus, researchers have been using adaptive technologies to effectively allocate computational resources. In the temporal dimension, existing works [10, 22–24] have mainly used the Courant–Friedrichs–Lewy condition to select optimal time steps, on the premise of maintaining the simulations’ numerical stability. In the following section, we discuss, in detail, adaptive techniques in the spatial domain.

Using different-size particles to discretize the simulated areas and dynamically adjust the particles’ radii during simulations enables adaptive allocation of computational resources to visually important areas. Keiser et al. [25] introduced a virtual particle-based multi-resolution coupling method to effectively split or merge particles, which increased the simulation speed up to six-fold. Adams et al. [5] proposed a method based on skeleton extraction, and then used coarse particles near the fluid skeleton, while using fine particles in areas far away from the skeleton, which improved the efficiency by three to eight times. Zhang et al. [26] implemented adaptive fluid sampling in a graphics processing unit (GPU)-based SPH framework. Orthmann and Kolb [27] used fine particles to conduct high-resolution simulations at the boundary of a multiphase flow, which improved the simulation accuracy. Winchenbach et al. [28] proposed an improved particle-splitting method that significantly improved stability during the particle-splitting process. Zhang et al. [29] dynamically refined the target computational domain by capturing the boundary of the simulated fluid, and proposed a particle splitting/merging criterion to avoid chain reactions during splitting/merging. Recently, Winchenbach and Kolb [30] derived a discretized objective function to adaptively adjust the particles’ radii in very high volume ratio scenarios (i.e., 1:1000000, or higher).

In Eulerian fluid simulation methods, adaptive remeshing can effectively improve the computational efficiency. Nakanishi et al. [31] proposed an adaptive PIC solver based on the radial basis function finite difference method to dynamically build a quadtree/octree in a narrow band near the liquid interface, maintaining the stability of the simulated system while reducing numerical dissipation and improving simulation accuracy. Xiao et al. [32] proposed an adaptive staggered tilted grid for incompressible flow simulations. Compared with a uniform Cartesian grid, adaptive grids can better discretize the complex simulation space, improving the simulation accuracy.

In addition to the field of computer graphics, some researchers in the field of computational physics have committed to the study of adaptive fluid simulation technologies. Qiang and Gao [11] proposed a method to adaptively determine the smoothing length of particles by combining changes in the particles’ density and the number of neighbors, but the iterative process for finding solutions is rather time-consuming. Yang and Kong [33] determined the particle smoothing length adaptively, using various physical properties of the neighboring particles, such as density and pressure, but the stability of this method is poor, especially in the cases of violent fluid movements.

## Methods

### Classical SPH

As a Lagrangian method, the SPH method seeks to discretize the simulated fluid into a collection of particles in space. Accounting for inter-particle interactions and external forces, the fluid particles’ dynamics obey Newton’s second law. The motion of all the fluid particles constitutes the overall motion of the fluid. During a simulation, the physical properties of the fluid particles, such as the density and force, are calculated by interpolation [3]:

1 $$A\left({x}_i\right)={\sum}_j{m}_j\frac{A_j}{\rho_j}W\left({x}_i-{x}_j,h\right)$$

where *A* represents the physical quantity to be computed, *x* represents the position of the particle, *i* and *j* denote the index of the particle, *m* is the mass of the particle, *ρ* is the particle density, *W* is the interpolation kernel, and *h* is the smoothing length of the particle.

In the interpolation calculation, the neighboring particles of a particle are determined by its smoothing length. The criterion for determining that particle *j* is a neighbor of particle *i* is that the Euclidean distance between particles *i* and *j* is less than the smoothing length *h* of particle *i*. When particle *j* is the neighbor of particle *i*, it influences the computation of the physical quantity of particle *i* through the above equation. Typically, the smoothing lengths are the same for all particles.

Before calculating the forces on a particle, it is necessary to calculate an important physical quantity, namely the density of particles. Based on Eq. 1, the density of particle *i* can be calculated as follows:

2 $${\displaystyle \begin{array}{c}\rho \left({x}_i\right)={\sum}_j{m}_jW\left({x}_i-{x}_j,h\right)\end{array}}$$

After the particle density is obtained, the forces acting on the particles can be calculated. The forces acting on a particle generally include pressure, viscosity, and external forces. The pressure and viscosity forces are calculated as follows:

3 $$\begin{array}{c}f_i^{pressure}=-\sum\limits_jm_j\frac{p_j}{\rho_j}\nabla W\left(x_i-x_j,h\right)\end{array}$$

4 $$\begin{array}{c}f_i^{vis\cos ity}=\mu\sum\limits_jm_j\frac{v_j}{\rho_j}\nabla^2W\left(x_i-x_j,h\right)\end{array}$$

where *p* is the pressure, *v* is the velocity, and *μ* denotes the viscosity coefficient. ∇ and ∇^2^ are the gradient operator and the Laplacian operator, respectively.

The external force usually refers to gravity; therefore, the total force on particle *i* is:

5 $${\displaystyle \begin{array}{c}{f}_i={f}_i^{pressure}+{f}_i^{vis\cos ity}+{\rho}_ig\end{array}}$$

Based on the above physical quantities, the acceleration and displacement of particles are calculated as follows:

6 $${\displaystyle \begin{array}{c}{a}_i=\frac{d{v}_i}{dt}=\frac{f_i}{\rho_i}\end{array}}$$

7 $${\displaystyle \begin{array}{c}\varDelta {x}_i=\left({v}_i+{a}_i\varDelta t\right)\varDelta t\end{array}}$$

where *a* represents acceleration and *t* is time.

The entire simulation process is summarized in Algorithm 1.

**Algorithm 1:**
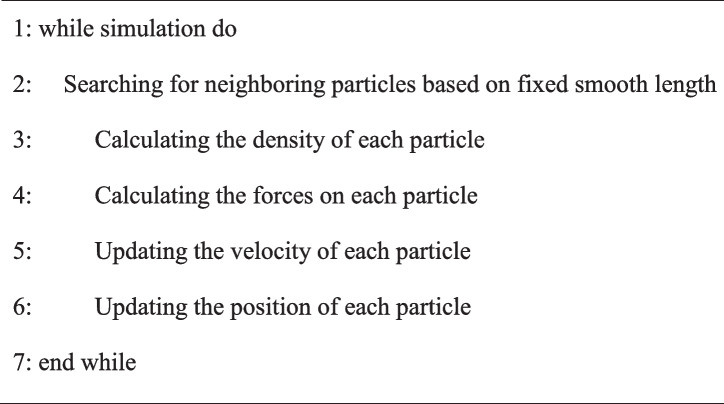
**Overview of traditional SPH method**

### Overview of our method

**Fig. 1:**
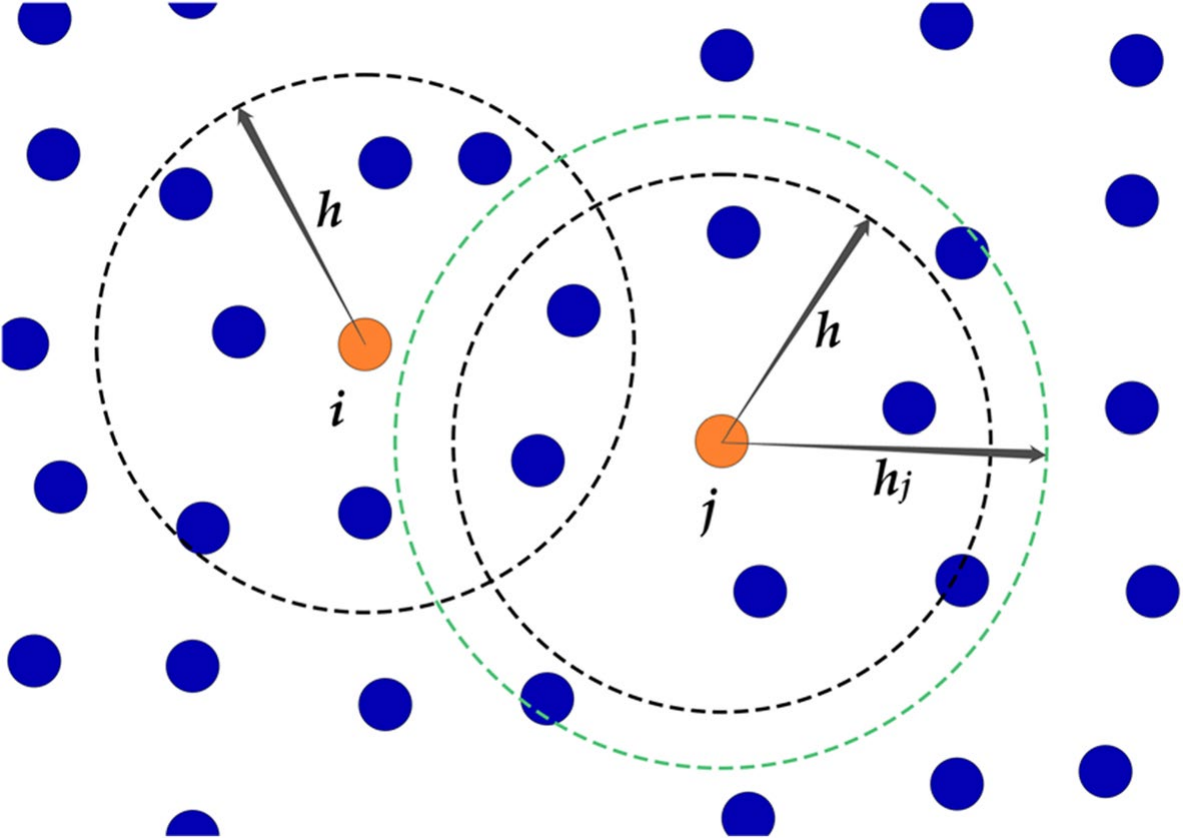
Illustration of the variable smoothing length concept. The smoothing length of particle *i* is the default value *h* owing to the uniform distribution of particles around it, while the smoothing length of particle *j* has to be expanded from *h* to *h*_*j*_ to maintain sufficient number of neighbors, owing to the sparse distribution of neighboring particles

In the SPH approach, physical quantities are discretely stored in Lagrangian fluid particles. During the simulation, physical quantities, such as a particle’s density and velocity, are interpolated based on their values for the particle’s neighbors. The number of neighboring particles is determined by the smoothing length, which is typically constant. A fixed smoothing length can cause numerical instability because it tends to take fewer neighbors for interpolation at splashes and surfaces. Variable smoothing-length methods [11, 33], in contrast, adaptively determine the smoothing length to maintain a constant number of neighboring fluid particles for interpolation (Fig. 1); however, their computational complexity is typically much higher, owing to their use of iterative optimization.

Aiming to improve the adaptiveness and efficiency of existing methods [11, 33], in this paper, we propose a variable smoothing-length method based on a non-iterative solution. The novel method first defines a control equation that assumes the mass of neighboring particles to be static, and then deduces the variable smoothing length in a non-iterative manner. By adjusting the number of neighboring particles directly and in a physically based manner, our method performs better in terms of the interpolation accuracy, and is more efficient because it only requires a non-iterative process to find the solution. Algorithm 2 provides an overview of our method, where the bold statements represent our improvements over the classical SPH method.

**Algorithm 2:**
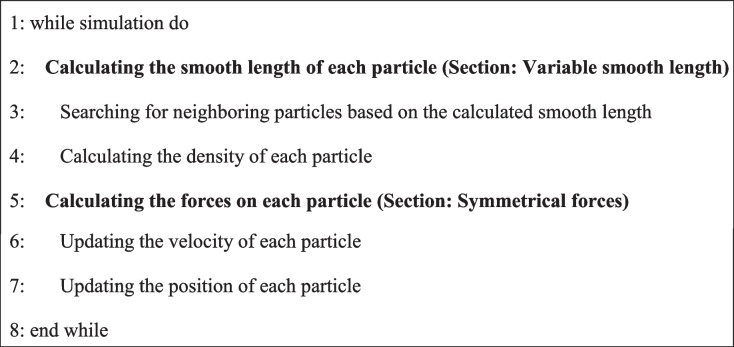
**Overview of our method**

### Variable smoothing length

The main objective of our method is to adaptively maintain the number of neighbors constant. Because the mass of a fluid particle remains unchanged, our objective can be reformulated as keeping the total mass [34] *m*_*sum*_ of the particles in the neighborhood unchanged.

Suppose *h*_*i*_ is the smoothing length of particle *i*, and *ρ*_*i*_ is its density; then, the total mass *m*_*sum*_ within its neighborhood is

8 $${\displaystyle \begin{array}{c}\sigma {h}_i^D{\rho}_i={m}_{sum}\end{array}}$$

where *D* is the dimension of the simulated system. Here, *σ* = *π* when *D* = 2, and *σ* = 4/3*π* when *D* = 3.

Because the mass is conserved in our simulations, *m*_*sum*_ does not depend on time. Differentiating the above volume-density equation (i.e., Eq. 8) with respect to time *t* yields the following equation:

9 $${\displaystyle \begin{array}{c}\sigma \left(D{h}_i^{D-1}\frac{\partial {h}_i}{\partial t}{\rho}_i+{h}_i^D\frac{\partial {\rho}_i}{\partial t}\right)=0\end{array}}$$

After eliminating the common factor $\sigma {h}_i^{D-1}$, Eq. 9 can be further simplified into

10 $${\displaystyle \begin{array}{c}D\frac{\partial {h}_i}{\partial t}{\rho}_i+{h}_i\frac{\partial {\rho}_i}{\partial t}=0\end{array}}$$

By rearranging Eq. 10 and dividing both sides by *Dρ*_*i*_, we finally obtain

11 $${\displaystyle \begin{array}{c}\frac{\partial {h}_i}{\partial t}=-\frac{1}{D}\frac{h_i}{\rho_i}\frac{\partial {\rho}_i}{\partial t}\end{array}}$$

The above equation can also be expressed as

12 $${\displaystyle \begin{array}{c}\frac{d{h}_i}{dt}=-\frac{1}{D}\frac{h_i}{\rho_i}\frac{d{\rho}_i}{dt}\end{array}}$$

Equation 12 establishes the relationship between the smoothing length and density and shows how they are connected to each other. Suppose the smoothing length and density of particle *i* are to be changed to *h*′_*i*_ and *ρ*′_*i*_ respectively after a time step *dt* (i.e., *dh*_*i*_ = *h*′_*i*_ − *h*_*i*_ and *dρ*_*i*_ = *ρ*′_*i*_ − *ρ*_*i*_); then, we have

13 $${\displaystyle \begin{array}{c}\frac{h_i^{\prime }-{h}_i}{h_i}=-\frac{1}{D}\frac{\rho_i^{\prime }-{\rho}_i}{\rho_i}\end{array}}$$

The above equation can be rearranged to

14 $${\displaystyle \begin{array}{c}\frac{h_i^{\prime }}{h_i}=1-\frac{1}{D}\left(\frac{\rho_i^{\prime }}{\rho_i}-1\right)\end{array}}$$

By multiplying both sides of the equation by *h*_*i*_, we get

15 $${\displaystyle \begin{array}{c}{h}_i^{\prime }=\left[1-\frac{1}{D}\left(\frac{\rho_i^{\prime }}{\rho_i}-1\right)\right]{h}_i\end{array}}$$

In simulations, numerical errors inevitably occur, which causes the distribution of particles to become uneven and causes density changes. Our aim is to keep the density of particles stable by adjusting the smoothing length of the particles. Therefore, the initial density of the particles (i.e., *ρ*_*i*_) in the above formula can be set as the net density value *ρ*_0_, which is usually 1000 kg/m^3^. Finally, we obtain the following equation to compute the new smoothing length of particle *i* based on the density change:

16 $${\displaystyle \begin{array}{c}{h}_i^{\prime }=\left[1-\frac{1}{D}\left(\frac{\rho_i^{\prime }}{\rho_0}-1\right)\right]{h}_i\end{array}}$$

where the current density ${\rho}_i^{\prime }$ of particle *i* is computed by interpolation from its neighboring particles [3, 9] using Eq. 2.

Using Eq. 16, the smoothing length is adaptively adjusted. For ${\rho}_i^{\prime }>{\rho}_0$ (i.e., when the interpolated density is too high and there are too many particles in the neighborhood, we attempt to correct the error by decreasing the smoothing length based on Eq. 16. In contrast, for ${\rho}_i^{\prime }<{\rho}_0$, we increase the smoothing length accordingly. Note that for ${\rho}_i^{\prime }={\rho}_0$, we have ${h}_i^{\prime }={h}_i$ and no correction is required.

In addition, a significant advantage of Eq. 16 is that its calculation is simple and direct, and no iterative solution is needed. As will be shown in the Results and Discussion section, the computational cost of adjusting the smoothing length in our method is almost negligible compared with the time-consuming step of searching for nearest neighbors.

### Symmetrical forces

To ensure the stability of the simulated system, the forces applied to the particles must satisfy Newton’s third law; that is, the force *f*_*ij*_ exerted by particle *i* on particle *j* should be equal to the force *f*_*ji*_ exerted by particle *j* on particle *i*, but in the opposite direction.

According to the proposed variable smoothing-length algorithm, each particle is assigned a unique smoothing length, which may cause instability. Suppose the smoothing lengths of particles *i* and *j* are *h*_*i*_ and *h*_*j*_ respectively, *h*_*ij*_ is the larger value of *h*_*i*_ and *h*_*j*_, and the Euclidean distance between the two particles is *d*_*ij*_. When *h*_*i*_ > *d*_*ij*_ > *h*_*j*_, particle *j* is considered to be the neighbor of particle *i*, and forces generated by particle *j* are applied to particle *i*. However, particle *i* is not considered as the neighbor of particle *j* in this case, and no force is exerted on particle *j* by particle *i*. As a result, the interaction between these two particles is asymmetrical, which does not conform to Newton’s third law.

To solve the above problem, inspired by the work in refs. [5, 9], we adopted a very effective strategy to form symmetrical forces between any pair of interacting particles. For particles *i* and *j*, if and only if *d*_*ij*_ < *h*_*ij*_, it is determined that particles *i* and *j* are neighbors. The interpolation weight is taken as the average of the weight obtained by taking *h*_*i*_ and *h*_*j*_ as the smoothing length. Specifically, the pressure and viscosity forces between the particles can be obtained using the following equations:

17 $${\displaystyle \begin{array}{c}\begin{array}{l}{f}_i^{pressure}=-{V}_i\sum\limits_j{V}_j\left({P}_i+{P}_j\right)\nabla {W}_{ij}\\ {}\nabla {W}_{ij}=\left(\nabla W\left({x}_i-{x}_j,{h}_i\right)+\nabla W\left({x}_i-{x}_j,{h}_j\right)\right)/2\end{array}\end{array}}$$

18 $${\displaystyle \begin{array}{c}\begin{array}{l}{f}_i^{viscosity}=\mu {V}_i\sum\limits_j{V}_j\left({v}_j-{v}_i\right){\nabla}^2{W}_{ij}\\ {}{\nabla}^2{W}_{ij}=\left({\nabla}^2W\left({x}_i-{x}_j,{h}_i\right)+{\nabla}^2W\left({x}_i-{x}_j,{h}_j\right)\right)/2\end{array}\end{array}}$$

where *V*_*i*_ = *m*/*ρ*_*i*_, *P*_*i*_ = *k*(*ρ*_*i*_/*ρ*_0_ − 1) and *k* is a gas constant that depends on temperature [3].

## Results and discussion

In this section, we present the experimental results of our proposed variable smoothing length SPH method (VSLSPH), and compare them with those obtained using several state-of-the-art methods, including WCSPH [14], PCISPH [7], IISPH [8], and DFSPH [9, 10]. All of the reported experiments were implemented using the open-source SPH library SPlisHSPlasH [35] on the same platform with an Intel i7-10700F CPU and 16 GB RAM.

### Variable smooth length of VSLSPH

**Fig. 2:**
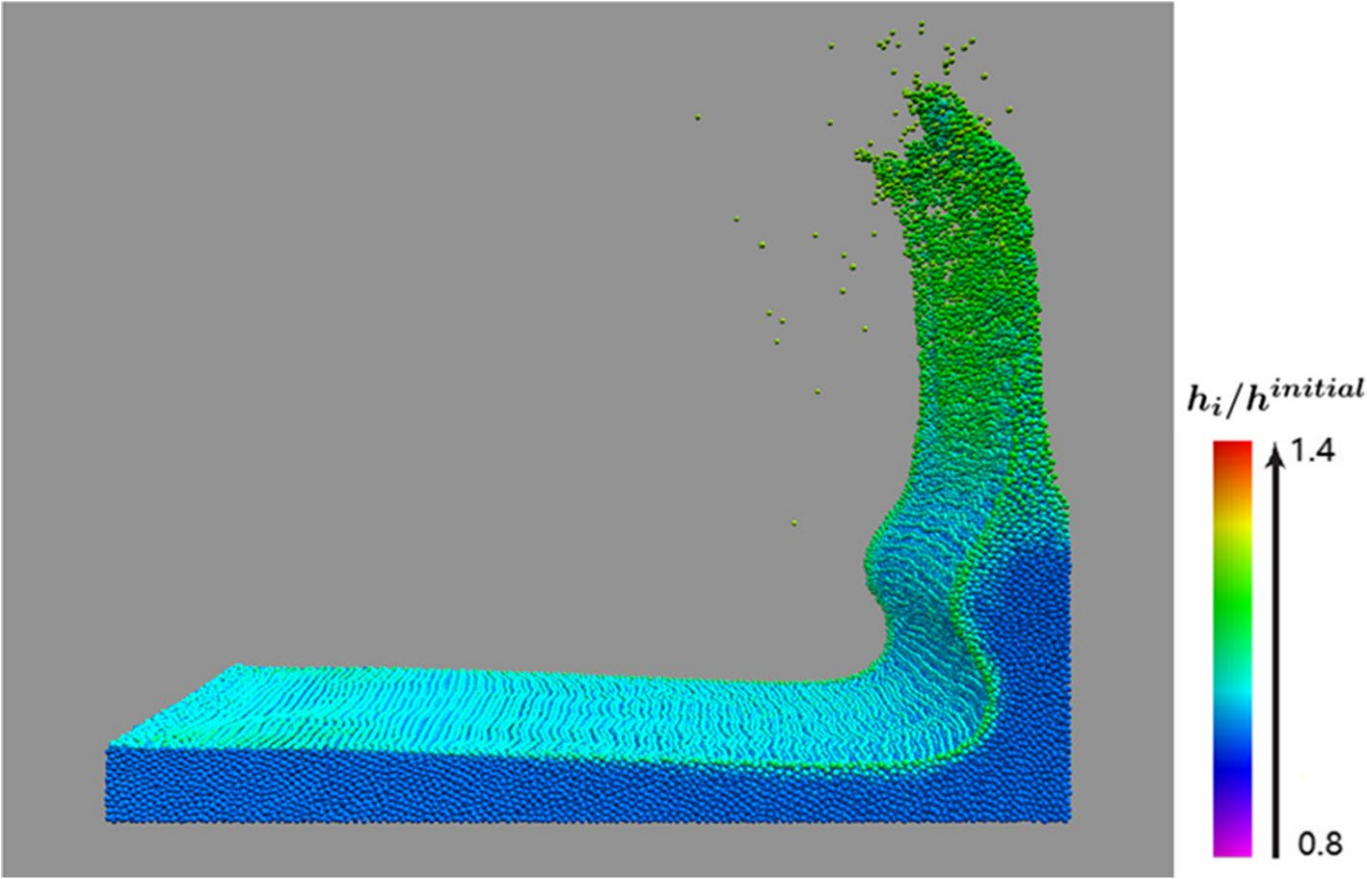
Simulation of a 3D dam-break scene using VSLSPH (131328 particles), where the smoothing lengths of particles are shown by different colors, ranging from purple to red

Our proposed VSLSPH method is advantageous for improving the accuracy of fluid simulations. Figure 2 shows the result of a three-dimensional (3D) dam break simulation, where particles with different smoothing lengths are shown with different colors. After applying our method, the smoothing lengths of individual particles were adjusted adaptively. Evidently, the smoothing lengths of the particles near the surface was clearly longer than for those in the liquid bulk, and particles in the splash area had even longer smoothing lengths. If required, more particles can be included in the interpolation using our method, yielding even higher simulation accuracies.

### Number of neighbors

**Fig. 3:**
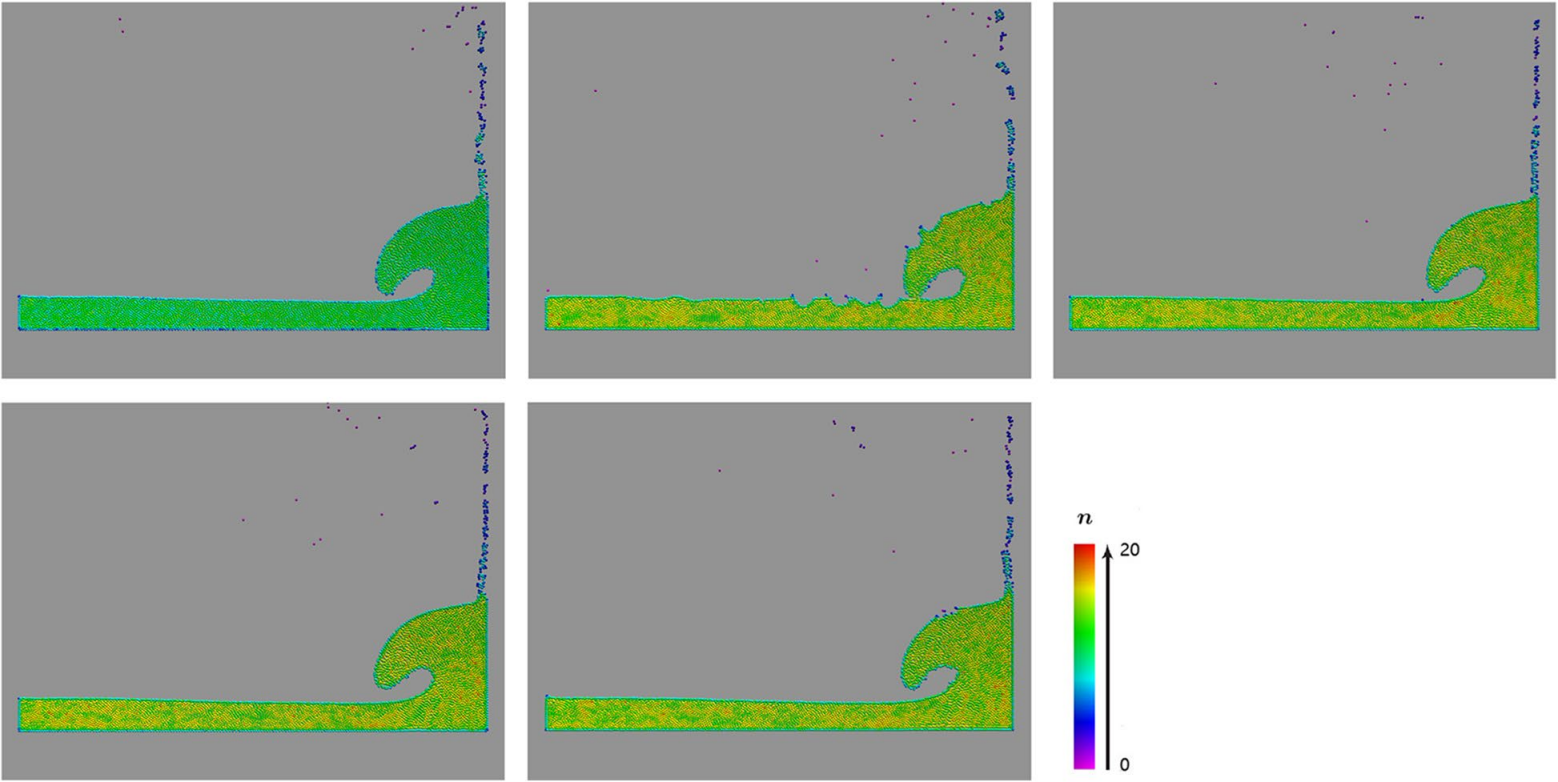
Simulations of a 2D dam-break scene (5476 particles) using different methods, where the number of neighbors per particle is shown by different colors, ranging from purple to red. Top: VSLSPH (the proposed method), WCSPH [14], and PCISPH [7]. Bottom: IISPH [8] and DFSPH [9, 10]

Maintaining the number of neighboring particles within a reasonable and fixed range is key to ensuring stable SPH simulations. Figure 3 shows the simulation results of a two-dimensional (2D) dam-break scene using the VSLSPH, WCSPH, PCISPH, IISPH, and DFSPH methods, where particles with different numbers of neighbors are plotted using different colors. Evidently, the proposed method yielded the most satisfactory result, because the color distribution is very uniform, which means that the number of neighbors per particle remained very stable throughout the system. In contrast, the color distributions for the other methods are very heterogeneous, implying significant variation in the number of neighbors, which can easily cause numerical instabilities.

**Fig. 4:**
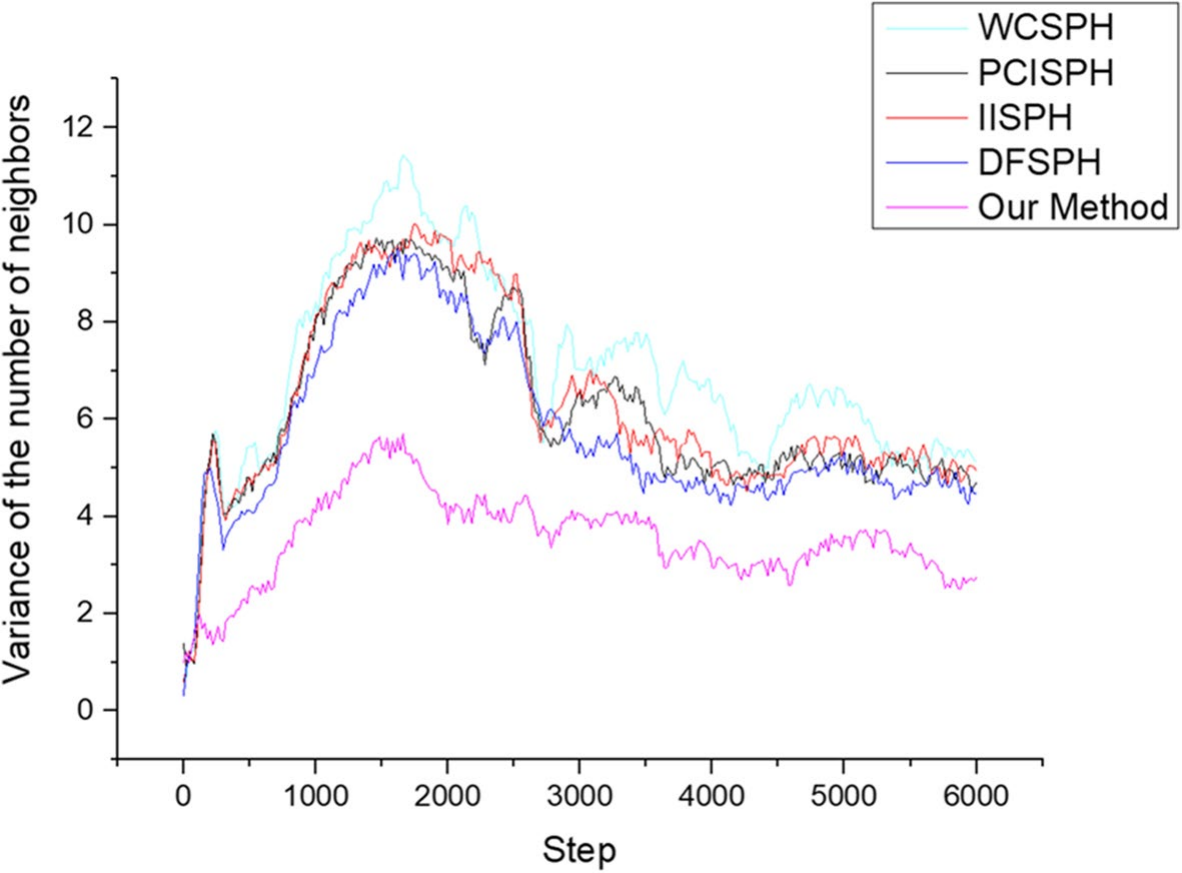
Variation in the number of neighbors

To further demonstrate the effectiveness of our method, we quantitatively compared the variance in the number of neighboring particles during the entire simulation, and the results are shown in Fig. 4. The average variance associated with the WCSPH method was the largest, implying significant changes in the number of particle neighbors. The other three methods that used a fixed smoothing length, namely the PCISPH, IISPH, and DFSPH methods, performed slightly better than the WCSPH method, but performed significantly worse than our proposed VSLSPH method, suggesting that our variable smoothing-length scheme effectively maintained the number of neighbors.

**Fig. 5:**
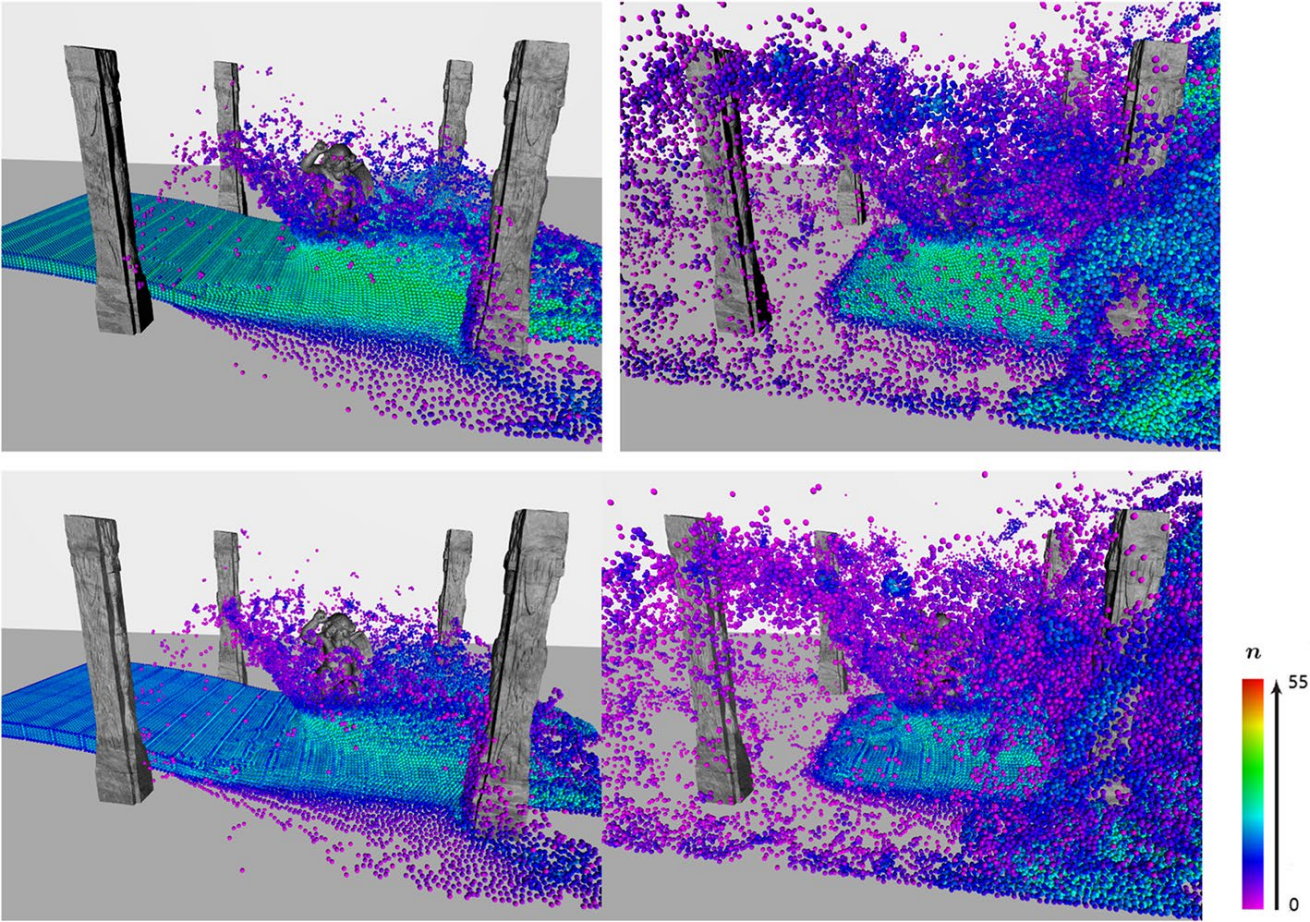
Simulation of water flow impacting a sculpture in ruins using the VSLSPH (top) and DFSPH [9, 10] (bottom) methods, with 20000 particles, where the number of neighbors per particle is shown by different colors, from purple to red. In the DFSPH case, many of the particles at the splash site are purple and the ones at the fluid surface are blue, while in the VSLSPH case, particles at the splash site are blue and the ones at the fluid surface are green, showing a better distribution of the number of neighbors

Figure 5 shows the simulation results of the water flow impacting a sculpture in ruins, performed using the DFSPH method [9, 10] and the proposed VSLSPH method. In the DFSPH case, most of the particles at the splash site are purple and those at the fluid surface are blue, implying that the number of particle neighbors at these sites is too low. In the VSLSPH case, the color of many particles at the splash site changed from purple to blue, and the color of the particles at the fluid surface changed from blue to green. In other words, the proposed method adaptively adjusted the smoothing length to obtain a more stable number of neighbors.

### Density

**Fig. 6:**
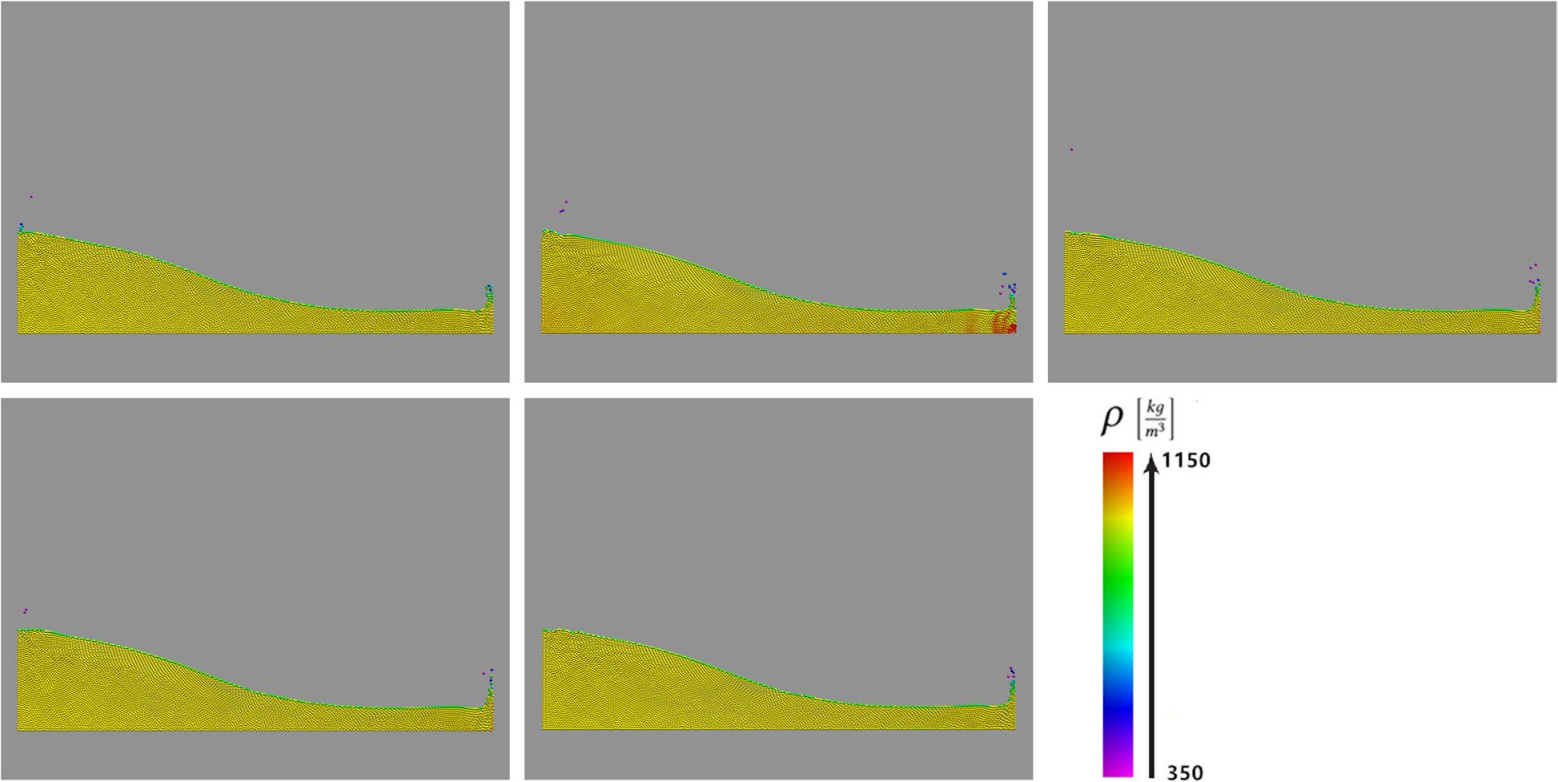
Fluid density fields of a 2D dam-break scenario simulated using different SPH-based algorithms (5476 particles). Top: VSLSPH (currently proposed), WCSPH [14], and PCISPH [7] methods; Bottom: IISPH [8] and DFSPH [9, 10] methods

As discussed, density is a very important physical quantity in SPH fluid simulations and is involved in many interpolation calculations; therefore, an important guarantee for improving the simulation accuracy is keeping the density of the entire fluid field stable. Figure 6 shows the fluid density field for a 2D dam-break scenario, simulated using different SPH-based algorithms. Because the WCSPH method [14] simply used a state equation to calculate the pressure, the incompressibility of the fluid was difficult to guarantee, resulting in a very uneven distribution of the density field. The VSLSPH method, on the other hand, yielded a uniform density field using the proposed variable smoothing-length scheme, which was competitive with the results of the algorithms using iterative density corrections, such as the PCISPH [7], IISPH [8], and DFSPH [9, 10] methods. Furthermore, as we show below, the simulation efficiency of the VSLSPH method was much higher than of the methods using iterative density corrections.

### Simulation efficiency

In this section, we compare the simulation efficiency of the VSLSPH method with that of related methods, using the 3D dam-break scenario in Fig. 2 as an example. The overall fluid simulation time was 5 s, and the number of particles was 131328. Table 1 lists some parameters, where ∆*t* is the time step, *t*_*sim*_ is the average time cost per step, *T*_*sim*_ is the time cost of the entire simulation (i.e., *T*_*sim*_ = 5.0/∆*t* ∗ *t*_*sim*_), *t*_*ns*_ is the average time cost of neighbor-searching per step, and *speedup* is the speedup ratio of various algorithms over the entire simulation time *T*_*sim*_, compared with the WCSPH method.

**Table 1:** Comparison of the simulation efficiency

Method	*Δt* (s)	*t*_*sim*_ (ms/step)	*T*_*sim*_ × 10^6^ (ms)	*t*_*ns*_ (ms/step)	Speedup
WCSPH	0.00002	75.704	18.907375	31.5168	–
PCISPH	0.00030	152.793	2.547075	36.4349	7.42
IISPH	0.00100	448.026	2.240130	33.5886	8.44
DFSPH	0.00150	306.114	1.020890	34.1696	18.52
VSLSPH	0.00150	271.625	0.909942	222.2490	20.78

Although the average single-step simulation cost using the WCSPH method is the lowest, its time step is also the smallest to maintain the numerical stability, resulting in the highest overall simulation time at the end. Compared with the three methods based on iterative density corrections (i.e., the PCISPH [7], IISPH [8], and DFSPH [9, 10] methods), the single-step simulation time cost of the VSLSPH method ranked in the middle, but its time step could be as large as that of the DFSPH method, enabling the lowest time cost for the entire simulation. As a result, the VSLSPH method was the most efficient, with 20-fold higher efficiency than the WCSPH method.

### Additional rendering results

**Fig. 7:**
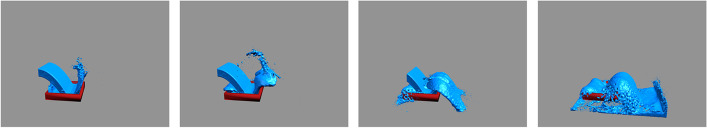
Visual simulation of a fluid pouring into a water tank and gradually filling a container, using the VSLSPH method (10000 particles)

**Fig. 8:**
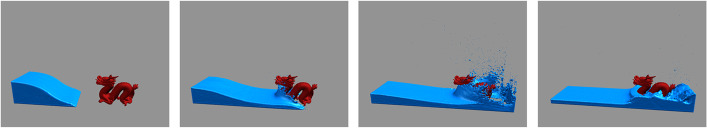
Visual simulation of a fluid scouring a dragon-shaped obstacle, using the VSLSPH method (117649 particles)

**Fig. 9:**
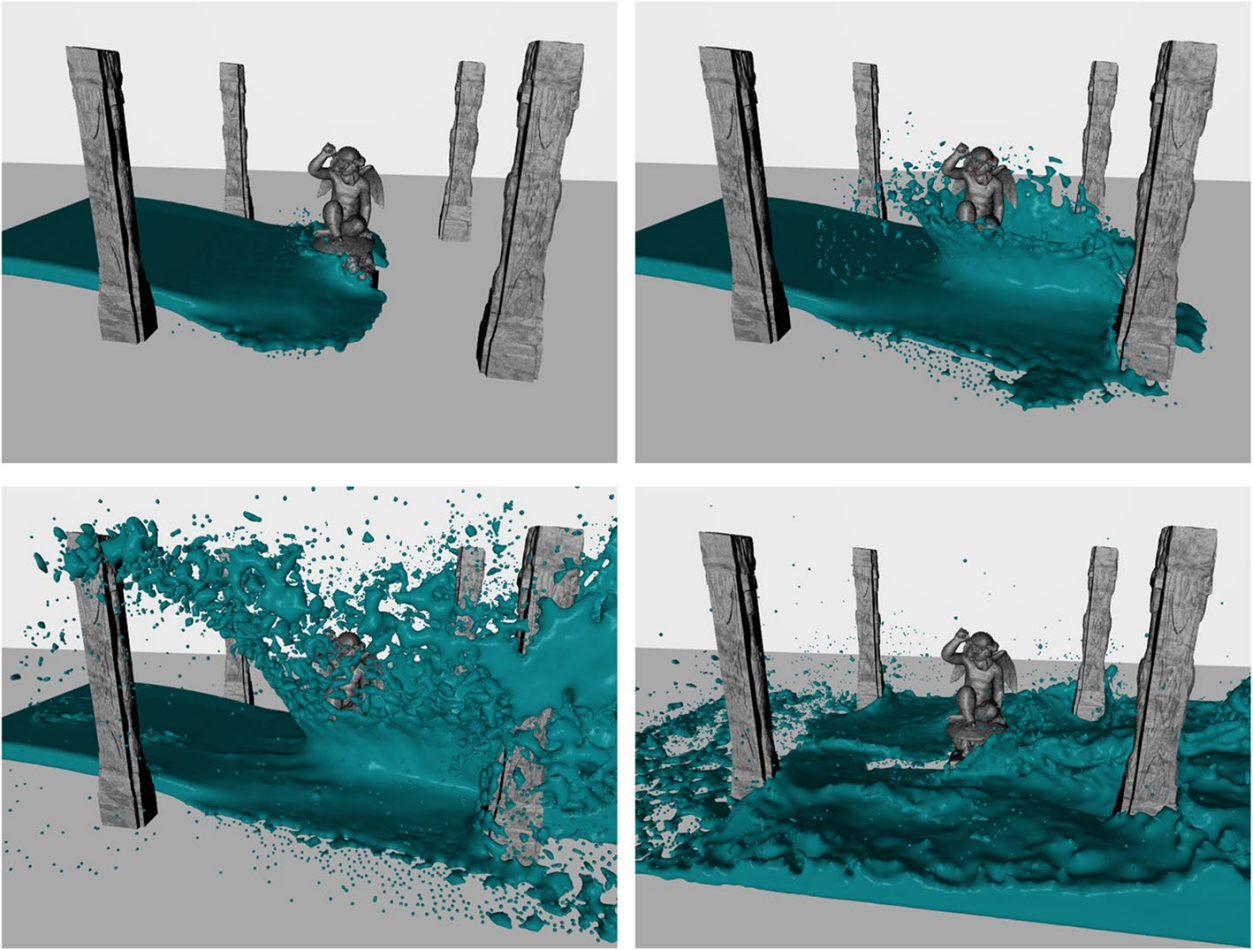
Visual simulation of a fluid flooding the ruins, using the VSLSPH method (200000 particles)

In addition to the above simulation results, which were specifically used for analysis and comparison, we also used the VSLSPH method to simulate and render additional fluid animations, to further demonstrate its effectiveness. Figure 7 shows the effect of fluid poured into a water tank and gradually filling a container. Figure 8 shows the effect of water scouring a dragon-shaped obstacle, while Fig. 9 shows the rendering result for the scenario in which a fluid flooded the ruins, which is also shown in the particle view in Fig. 5. The numbers of particles in the above three scenarios were 10000, 117649, and 200000, respectively. The above rendering results reveal very rich fluid details, including splashes, water sprays, and breaking waves, while maintaining the stability of the simulations.

## Discussion

In computational physics, methods such as the one proposed by Qiang and Gao [11] also used variable smoothing length for SPH fluid simulations. However, these methods typically involve complex numerical solutions for high accuracy, and are mainly tested in 2D simulations, which are not suitable for 3D fluid animations in computer graphics, as considered in this study, because they have higher requirements on the simulation speed. Moreover, the method proposed by Qiang and Gao [11] is only suitable for simulating physical phenomena such as explosions and shock waves.

As shown in Table 1, for the three algorithms based on iterative density corrections (i.e., the PCISPH [7], IISPH [8], and DFSPH [9, 10] methods), the average time cost of neighbor search accounted for approximately 7%–25% (estimated as *t*_*ns*_/*t*_*sim*_ ∗ 100%) of the total cost of each step, and the main computational cost was the iterative optimization process for determining optimal density corrections. However, in the WCSPH and VSLSPH approaches, neighbor search incurred significant computational cost, reaching 81.82% for the VSLSPH method. The VSLSPH method uses variable smoothing length, and the commonly used fast neighbor-search methods (e.g., using auxiliary grids [1] or searching trees [36]) are no longer applicable; thus, neighbor search becomes very time-consuming in the VSLSPH approach. The excessive computational overhead of neighbor search is a limitation of our approach, and we leave it as an important problem to be solved in the future.

## Conclusions and future work

In this paper, a non-iterative SPH fluid simulation method, called VSLSPH, was proposed for adaptively adjusting the smoothing length of particles, allowing to effectively maintain a stable number of particle neighbors (especially on the fluid surface) and thus improving the simulation accuracy. In contrast to the existing methods based on iterative optimization to correct the particle density, our method establishes a direct connection between the smoothing length and density change, resulting in a very low computational cost and high simulation efficiency.

As shown in our experiments, neighbor searching constitutes most of the computational overhead in the VSLSPH approach. One of the main tasks in the future will be to develop a fast neighbor-search algorithm for variable smoothing lengths, to improve the overall simulation efficiency of the VSLSPH method. In addition, the GPU implementation of our algorithm is an interesting topic for future work.

## Data Availability

The source code is available by emailing to hongshul@foxmail.com for non-commercial usage.

## Abbreviations

SPH: Smoothed particle hydrodynamics
GPU: Graphics processing unit
VSLSPH: Variable smoothing length smoothed particle hydrodynamics
3D: Three-dimensional
2D: Two-dimensional

## References

[1] Koschier D, Bender J, Solenthaler B, Teschner M (2019). Smoothed particle hydrodynamics techniques for the physics based simulation of fluids and solids.

[2] Ihmsen M, Orthmann J, Solenthaler B, Kolb A, Teschner M (2014). SPH fluids in computer graphics.

[3] Müller M, Charypar D, Gross M (2003). Particle-based fluid simulation for interactive applications.

[4] Lyu HG, Sun PN, Huang XT, Zhong SY, Peng YX, Jiang T (2022). A review of SPH techniques for hydrodynamic simulations of ocean energy devices. Energies.

[5] Adams B, Pauly M, Keiser R, Guibas LJ (2007). Adaptively sampled particle fluids.

[6] Sun PN, Le Touzé D, Oger G, Zhang AM (2021). An accurate SPH Volume Adaptive Scheme for modeling strongly-compressible multiphase flows. Part 1: numerical scheme and validations with basic 1D and 2D benchmarks. J Comput Phys.

[7] Solenthaler B, Pajarola R (2009). Predictive-corrective incompressible SPH.

[8] Ihmsen M, Cornelis J, Solenthaler B, Horvath C, Teschner M (2014). Implicit incompressible SPH. IEEE Trans Vis Comput Graph.

[9] Bender J, Koschier D (2015). Divergence-free smoothed particle hydrodynamics.

[10] Bender J, Koschier D (2017). Divergence-free SPH for incompressible and viscous fluids. IEEE Trans Vis Comput Graph.

[11] Qiang HF, Gao W (2008). SPH method with fully variable smoothing lengths and implementation. Chin J Comput Phys.

[12] Khayyer A, Shimizu Y, Gotoh H, Nagashima K (2021). A coupled incompressible SPH-hamiltonian SPH solver for hydroelastic FSI corresponding to composite structures. App Math Model.

[13] Lyu HG, Sun PN, Huang XT, Chen SH, Zhang AM (2021). On removing the numerical instability induced by negative pressures in SPH simulations of typical fluid-structure interaction problems in ocean engineering. Appl Ocean Res.

[14] Becker M, Teschner M (2007). Weakly compressible SPH for free surface flows.

[15] Wu ML, Liu SG, Xu Q (2021). Improved divergence-free smoothed particle hydrodynamics via priority of divergence-free solver and SOR. Comput Anim Virtual Worlds.

[16] Yang T, Martin RR, Lin MC, Chang J, Hu SM (2007). Pairwise force SPH model for real-time multi-interaction applications. IEEE Trans Vis Comput Graph.

[17] Weiler M, Koschier D, Brand M, Bender J (2018). A physically consistent implicit viscosity solver for SPH fluids. Comput Graph Forum.

[18] Band S, Gissler C, Ihmsen M, Cornelis J, Peer A, Teschner M (2018). Pressure boundaries for implicit incompressible SPH. ACM Trans Graph.

[19] Bender J, Kugelstadt T, Weiler M, Koschier D (2019). Volume maps: an implicit boundary representation for SPH.

[20] Bender J, Kugelstadt T, Weiler M, Koschier D (2020). Implicit frictional boundary handling for SPH. IEEE Trans Vis Comput Graph.

[21] Gissler C, Peer A, Band S, Bender J, Teschner M (2019). Interlinked SPH pressure solvers for strong fluid-rigid coupling. ACM Trans Graph.

[22] Ihmsen M, Akinci N, Akinci G, Teschner M (2012). Unified spray, foam and air bubbles for particle-based fluids. Vis Comput.

[23] Schechter H, Bridson R (2012). Ghost SPH for animating water. ACM Trans Graph.

[24] He F, Zhang HS, Huang C, Liu MB (2022). A stable SPH model with large CFL numbers for multi-phase flows with large density ratios. J Comput Phys.

[25] Keiser R, Adams B, Dutré P, Guibas LJ, Pauly M (2006). Multiresolution particle-based fluids.

[26] Zhang YC, Solenthaler B, Pajarola R (2008). Adaptive sampling and rendering of fluids on the GPU.

[27] Orthmann J, Kolb A (2012). Temporal blending for adaptive SPH. Comput Graph Forum.

[28] Winchenbach R, Hochstetter H, Kolb A (2016). Constrained neighbor lists for SPH-based fluid simulations.

[29] Zhang K, Sun YJ, Sun ZG, Wang F, Chen X, Xi G (2022). An efficient MPS refined technique with adaptive variable-size particles. Eng Anal Bound Elem.

[30] Winchenbach R, Kolb A (2021). Optimized refinement for spatially adaptive SPH. ACM Trans Graph.

[31] Nakanishi R, Nascimento F, Campos R, Pagliosa P, Paiva A (2020). RBF liquids: an adaptive PIC solver using RBF-FD. ACM Trans Graph.

[32] Xiao YW, Chan S, Wang SQ, Zhu B, Yang XB (2020). An adaptive staggered-tilted grid for incompressible flow simulation. ACM Trans Graph.

[33] Yang XF, Kong SC (2019). Adaptive resolution for multiphase smoothed particle hydrodynamics. Comput Phys Commun.

[34] Springel V, Hernquist L (2002). Cosmological smoothed particle hydrodynamics simulations: the entropy equation. Mon Not R Astron Soc.

[35] GitHub - InteractiveComputerGraphics/SPlisHSPlasH: SPlisHSPlasH is an open-source library for the physically-based simulation of fluids. https://github.com/InteractiveComputerGraphics/SPlisHSPlasH. Accessed 24 Mar 2019

[36] Gong XF, Yang JM, Zhang SD (2016). A parallel SPH method with background grid of adaptive mesh refinement. Chin J Comput Phys.

